# Palatability and Acceptability of Flaxseed-Supplemented Foods in Children with Sickle Cell Disease

**DOI:** 10.3390/nu15051245

**Published:** 2023-03-01

**Authors:** Chinenye R. Dike, Jeffrey Lebensburger, Ciara Mitchell, Betty Darnell, Casey D. Morrow, Wendy Demark-Wahnefried

**Affiliations:** 1 Department of Pediatrics, Division of Pediatric Gastroenterology, Hepatology and Nutrition, University of Alabama at Birmingham, Birmingham, AL 35294, USA; 2 Department of Pediatrics, Division of Pediatric Hematology and Oncology, University of Alabama at Birmingham, Birmingham, AL 35294, USA; 3 Bionutrition Unit, Center for Clinical and Translational Science, University of Alabama at Birmingham, Birmingham, AL 35294, USA; 4 Department of Cell, Developmental and Integrative Biology, University of Alabama at Birmingham, Birmingham, AL 35294, USA; 5 Department of Nutrition Sciences, University of Alabama at Birmingham, Birmingham, AL 35294, USA

**Keywords:** flaxseed, diet, omega-3 fatty acids, sickle cell disease, children, pain

## Abstract

Omega-3 fatty acids (w3FAs) have demonstrated benefits in several inflammatory disease states; however, limited research has been conducted in sickle cell disease (SCD). While marine-based w3FAs are used, their strong odor and taste are a barrier to long-term use. Plant-based sources, especially those in whole foods, may circumvent this barrier. We tested whether flaxseed (rich source of w3FAs) was acceptable to children with SCD. A cross-sectional tasting trial of flaxseed added to baked products (cookies, pancakes, brownies) or to readily available foods (applesauce, pudding, yogurt) was conducted among 30 children (median age = 13 years) reporting to a clinic for routine follow-up, sick visits, or transfusion for SCD to determine acceptability. A food preference rank scale (1–7) was used to rank products based on taste, sight, smell, and texture. An average score for each product was computed. Children were also asked to rank their top three products. The top-ranked products were flaxseed baked in brownies and cookies and ground flaxseed added to yogurt. More than 80% of participants indicated willingness to be contacted for a follow-up study in which a flaxseed-supplemented diet would be evaluated for mitigation of SCD-associated pain. In conclusion, flaxseed-enriched products are palatable and acceptable in children with SCD.

## 1. Introduction

Sickle cell disease (SCD) is a chronic hemolytic condition that can lead to multi-organ damage [1]. There are about 300,000 neonates born worldwide every year with SCD, with millions of people affected by the disease [2]. In the United States, there are about 100,000 individuals living with this disease [2]. Peterson et al., on review of Healthcare Cost and Utilization Project (HCUP) databases, found that in children less than 18 years living in the United States with the HbSS genotype (severe type of SCD), there was an annual expenditure of USD 97.7 million for admissions through the ED [3]. SCD poses a substantial healthcare burden of approximately USD 2.4 billion annually in the United States [4]. In SCD, red blood cells undergo polymerization in the deoxygenated state, which produces oxidative stress and hemolytic episodes [5]. This intravascular hemolysis promotes inflammation and endothelial dysfunction [6]. The combination of polymerization, vaso-occlusion, and endothelial dysfunction caused by hemolysis and inflammation drives the adverse clinical outcomes of SCD [6]. Given this known pathogenesis, antioxidants that reduce stress and subsequent hemolysis and inflammation have been studied as interventions to decrease hemolytic episodes and improve clinical outcomes in SCD [5]. Some of these interventions include N-acetylcysteine [7], L-arginine [8], I-glutamine [9], zinc [10], fat-soluble vitamins [11,12], and omega-3 fatty acids [13,14].

Marine-based omega-3 fatty acids (found in fish oil) have been shown to decrease inflammation [15], rates of vaso-occlusive events [13,16], peak systolic cerebral artery blood velocity [17], and improve severe anemia and white cell count [13]. However, fish oil supplements are prone to lipid oxidation, which is responsible for the rancid taste and smell [18]. Fish oil supplements contain polyunsaturated fatty acids (PUFAs), particularly eicosapentaenoic acid (EPA) and docosahexaenoic acid (DHA). These PUFAs contain multiple double bonds that can undergo oxidation causing fishy odors and taste [19].

Fats, flavorings, and sweeteners may mask the intensity of the undesirable fishy taste but have little effect on lipid peroxidation and formation of free radicals [20]. Recent data suggest that highly flavored fish oil supplements and supplements made especially for children had significantly higher primary, secondary, and total oxidation levels than others, raising concerns regarding safety [21].

Given the benefits of omega-3 fatty acids in managing inflammation, but recognizing the side effect concerns for fish oil, we aimed to determine the acceptance and palatability of plant-based omega-3 fatty acids from flaxseed in children with SCD. We hypothesized that products with added flaxseed would be acceptable to a cohort of children with SCD.

## 2. Materials and Methods

### 2.1. Design/Participants

We performed a pilot cross-sectional study of children aged 5–18 years followed at the comprehensive sickle cell clinic at the Children’s Hospital of Alabama/University of Alabama at Birmingham (UAB). This study was approved by the UAB Institutional Review Board (IRB #300010072). We performed this study during sickle cell clinics for children aged 5–18 years with SCD. Participants were approached for enrollment discussion by the principal investigator (C.R.D). Signed informed consent, with or without assent, was obtained by C.R.D. Our inclusion criteria included children with SCD (all genotypes, including HbSS, HbSβ0 thalassemia, HbSC, and HbSβ+ thalassemia) aged 5–18 years old presenting to the SCD clinic or transfusion clinic at the Children’s Hospital of Alabama. We excluded participants with known allergy to flaxseed or known to be pregnant or lactating.

### 2.2. Materials

Flaxseed-added baked and raw products were designed by UAB Center for Clinical and Translational Science (CCTS)-registered dietitians (B.D. and C.M.), and the products were prepared by the metabolic kitchen cooks affiliated with the CCTS at UAB. The baked flaxseed products were cookies, pancakes, and brownies, while freshly ground flaxseed was added to yogurt, pudding, and applesauce. Individually wrapped servings were prepared to deliver 5 g of flaxseed per serving (1.1 mg of alpha-linolenic acid). Applesauce, pudding, yogurt, and pancakes were refrigerated until served, while cookies and brownies were kept at room temperature.

### 2.3. Data Collection

Participants were asked to complete a survey form adapted from Chen et al. [22] using Hedonic scales to measure flaxseed-containing food preferences. For this study, we asked participants to rank six flaxseed-containing products: brownies, cookies, pancakes, applesauce, pudding, and yogurt. The participants were asked to use a 1–7 visual analog scale (1: superbad, 2: really bad, 3: bad, 4: maybe good or maybe bad, 5: good, 6: really good and 7: super good) for four senses: appearance, smell, taste, and texture (Supplemental content 1). After providing scores for individual senses, participants were asked to rank each product from favorite to most disliked and pick their top three products (see attached survey form used-supplemental content). Finally, participants and their families were asked to indicate their interest in being contacted for participation in future studies that involved flaxseed feeding for at least 4 weeks. The parents or research personnel assisted younger children who could not complete the survey independently. Parents and children above 14 years were asked to rate the recipes (Supplemental content 2) from 1–10 based on ease to replicate recipe (1: very difficult to replicate to 10: very easy to replicate). Data were entered into an electronic capture software REDCap.

### 2.4. Statistical Considerations

This exploratory study was descriptive in nature; thus, we performed descriptive statistics of patient characteristics, including sex, age, and location of residence (urban, rural, or unsure). We also determined the mean scores with standard deviations (SDs) and standard errors of mean (SEMs) for appearance, smell, taste, and texture for each of the products. In addition, ease of product replication was also expressed as means with standard deviations. Proportions were used to represent participant’s willingness to participate in future flaxseed-supplemented trials. Microsoft excel version 2208 Build 16.0.15601.20446 64-bit© was used to calculate these mean scores, standard deviations, standard errors of means, medians, and interquartile ranges (IQRs). JMP 16PRO (Cary, NC, USA) was used for chi square testing for top-ranked products; a *p*-value of <0.05 was considered statistically significant.

## 3. Results

### 3.1. Cohort Characteristics

In total, 39 children with SCD were approached for enrollment over 10 business days in December 2022 (from 13 December to 31 December 2022). Eight children declined participation in the study. One child who agreed to participate did not come with a parent to the clinic visit and could not reach a caregiver by phone; therefore, consent was not possible. Thirty participants were consented and enrolled into the study.

Of the 30 participants enrolled, 15 were females and 15 were males. The median age of participants in this cohort was 13 years with IQR 5.75 (9.5–15.25). Fifteen reported living in an urban setting while nine resided rurally (six were unknown).

### 3.2. Outcomes

#### Product Preference Scores

- Appearance

**Figure 1:**
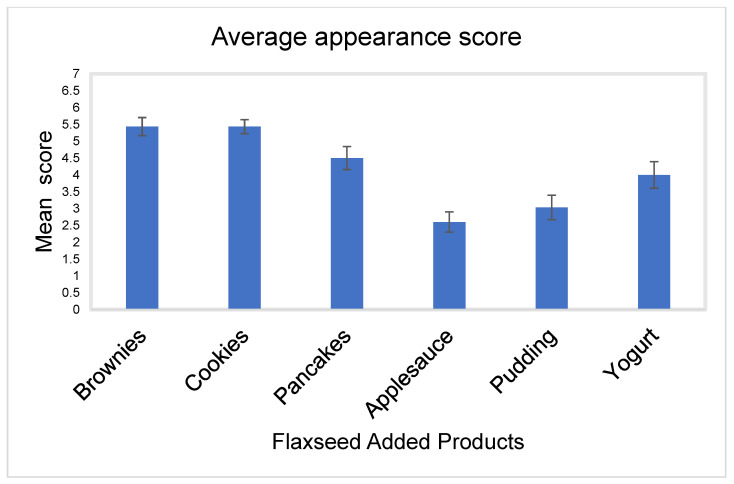
Bar chart showing the mean appearance score for all flaxseed products with standard errors of mean (SEMs).

Flaxseed-baked brownies and cookies had the highest mean scores for appearance, with mean scores of 5.4, with standard deviations of 1.5 and 1.1, and SEMs of 0.3 and 0.2, respectively. Pancakes followed with a mean appearance score of 4.5 ± 1.9, SEM 0.3, yogurt with a mean appearance score of 4 ± 2.2, SEM 0.4, pudding with a mean appearance score of 3 ± 2.0, SEM 0.4, and then applesauce with a mean appearance score of 2.6 ± 1.7, SEM 0.3 (Figure 1).

- Smell

**Figure 2:**
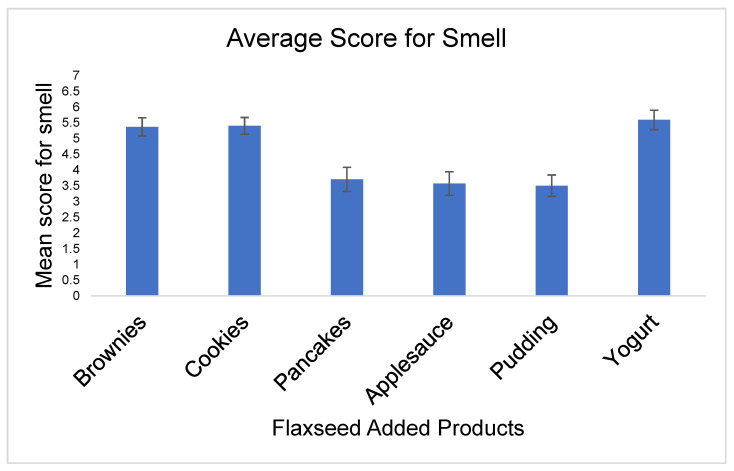
Bar chart of the mean score with SEM for smell for all flaxseed products.

Flaxseed added in yogurt had the highest average score for smell with a mean score of 5.6 ± 1.7, SEM 0.3. This was closely followed by flaxseed-baked cookies and brownies, with mean scores of 5.4 and standard deviations of 1.5 and 1.6, respectively, and SEM of 0.3, then pancakes with a mean score of 3.7 ± 2.1, SEM of 0.4, applesauce with mean score of 3.6 ± 2.0 and SEM 0.4, and then pudding with mean score of 3.5 ± 1.9 and SEM 0.3 (Figure 2).

- Taste

**Figure 3:**
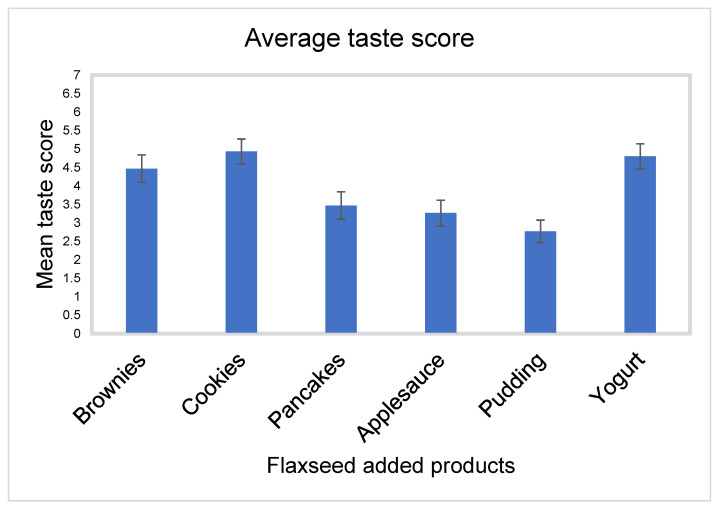
Mean score with SEM for taste for all flaxseed products.

Flaxseed-baked cookies had the highest mean score for taste, with a mean of 4.9 ± 1.8 and SEM 0.3. This was followed by flaxseed added in yogurt with a mean score of 4.8 ± 1.9 and SEM 0.3, then flaxseed-baked brownies with a mean score of 4.5 ± 2.0 and SEM 0.4, pancakes with mean score of 3.5 ± 2.0 and SEM 0.4, applesauce with mean score of 3.3 ± 1.9 and SEM 0.3, and then pudding with mean score of 2.8 ± 1.7 and SEM 0.3 (Figure 3).

- Texture

**Figure 4:**
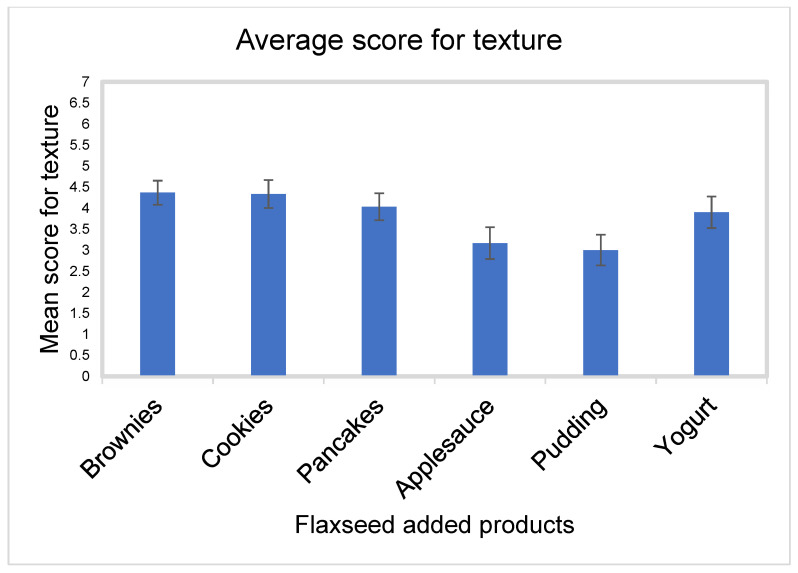
Mean score with SEMs for texture for all flaxseed products.

Flaxseed-baked brownies had the highest mean score for texture, with a mean score of 4.4 ± 1.6 and SEM 0.3. This was followed by flaxseed-baked cookies, with a mean score of 4.3 ± 1.8 and SEM 0.3, flaxseed-baked pancakes with mean score of 4.0 ± 1.8 and SEM 0.3, flaxseed-added yogurt with mean score of 3.9 ± 2.1 and SEM 0.4, flaxseed-added applesauce with mean score of 3.2 ± 2.1 and SEM 0.4, and then flaxseed added in pudding with mean score of 3.0 ± 2.0 and SEM 0.4 (Figure 4).

### 3.3. Other Outcomes

- Overall score

**Figure 5:**
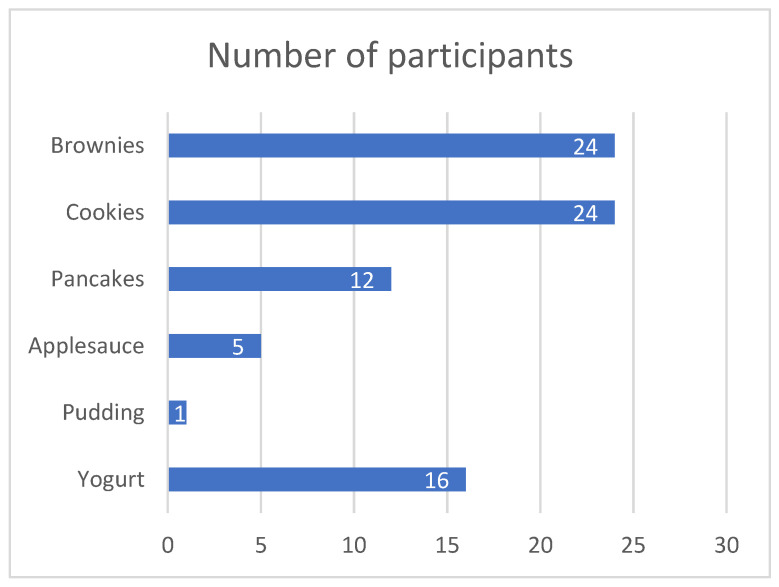
Bar chart of overall ranking of products.

Brownies were identified as the top-ranked overall product (~47%, *p* < 0.0001). The top-three-ranked products were brownies, cookies, and yogurt, which scored significantly higher than the other three products (*p* < 0.0001, Figure 5).

- Willingness to participate in future studies.

**Figure 6:**
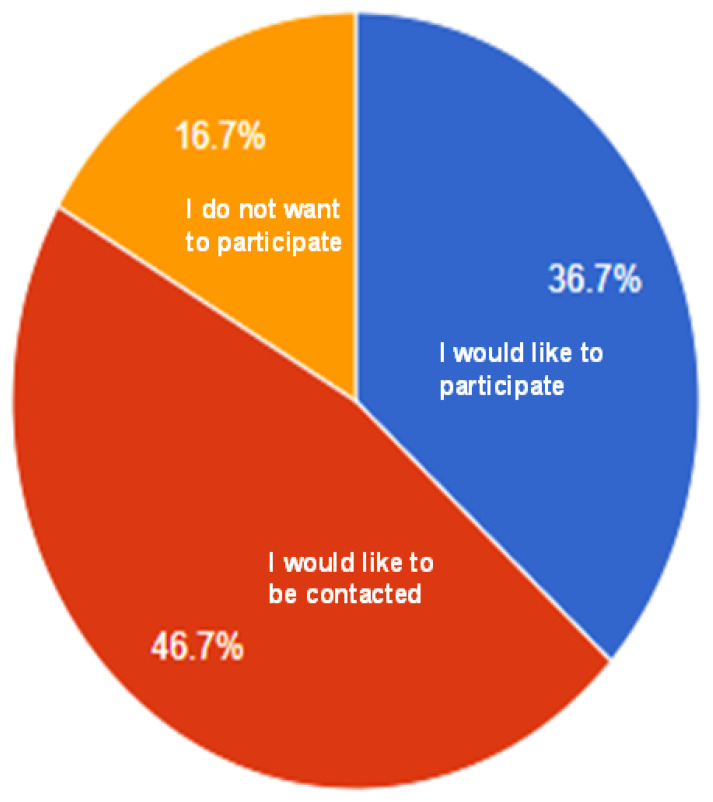
Pie chart of willingness to participate in future studies.

Twenty-five participants (83.3%) stated they would like to be contacted for a follow-up study that would involve eating flaxseed-added products for at least 4 weeks, indicating willingness to participate in future research (Figure 6). The mean score for ease of replication of the recipes was: brownies 8.7 ± 1.9, pancakes 7.8 ± 2.5, and cookies 7.7 ± 2.2. One of our participants takes fish oil capsules daily at home and preferred the taste of brownies, cookies, and pancakes to his daily fish oil supplements.

## 4. Discussion

Sickle cell clinical trials suffer from poor enrollment and retention as well as poor adherence to therapy [23]. Therefore, it is critical that sickle cell trials incorporate patient attitudes and beliefs about the acceptability of the intervention. Our study showed that flaxseed products are palatable and acceptable to a cohort of children with SCD. Importantly, our pilot study identified that > 80% of participants reported willingness to be contacted for a follow-up study that would involve ingesting flaxseed-added products for at least 4 weeks.

Marine-based omega-3 fatty acids have been shown to improve pain outcomes in patients with SCD [13] through a docosahexaenoic acid (DHA) increase in red blood cell flexibility [24]. Plant-based omega-3 fatty acid sources such as flaxseed are less studied than those from marine-based sources, because the conversion of alpha-linoleic acid to longer-chain fatty acids, eicosapentaenoic acid (EPA) and docosahexaenoic acid (DHA), may be rate limited, particularly in populations including Americans, who consume large amounts of omega-6 fatty acids. However, a study by Demark-Wahnefried and colleagues [25] found that prostate cancer patients supplemented with flaxseed for an average of 31 days experienced significant increases in erythrocyte membrane EPA (*p* = 0.005) compared to controls, whereas no increase in alpha- linolenic acid (ALA) content was observed. Similar results were found for the fatty acid content of the prostate (*p* = 0.010), and supplemented men also experienced significantly lower tumor proliferation. These findings suggest that the conversion of alpha-linoleic acid is effectively converted to longer-chain fatty acids such as EPA and, thus, may reduce pain just as effectively as fatty acids from marine sources. Flaxseed is also rich in fiber and has both antioxidant and anti-inflammatory properties [26]. The benefits of flaxseed have been reported in improving pain in patients with rheumatoid arthritis [27], modulating the gut microbiome, and decreasing the severity of type 2 diabetes assessed in a murine model [28].

Despite benefits observed with flaxseed feeding in adults, relatively few studies have been conducted in children, with one randomized controlled trial among 32 children with hypercholesterolemia showing no benefit of flaxseed vs. wheat flour on serum lipids after a 4-week period [29], and another 4-week trial among 72 children with obesity, randomized to either puffed wheat or flaxseed, showing no differences in adiposity, though children receiving flaxseed were found to have significantly less mental fatigue [30]. In contrast, an observational study of 73 patients (ages 7–24) with Familial Mediterranean Fever, an autosomal recessive disorder characterized by recurrent attacks of fever, serositis, and articular pain and fed a diet supplemented with vitamin D, curcumin, and flaxseeds reported significant improvement in clinical presentation, cognitive functions, c-reactive protein, and subjective wellbeing over a 6-month period [31]. However, in all this work, there is little reported regarding the acceptance of flaxseed. Our study revealed that in a cohort of children with sickle cell disease, flaxseed-added products were palatable and acceptable. Additionally, recipes presented to families were deemed relatively easy to replicate, and ground flaxseed could be added to readily available food, such as yogurt and applesauce.

The concept of personal agency over one’s environment has been implicated as a significant contributor to the formation of preferences [32]. Additionally, the knowledge of greater agency may affect personal preferences [32]. Furthermore, some implicit measures of external sound and smell stimuli have been shown to facilitate recovery after cognitive stress [33]. A study by Tonacci and colleagues also suggested that increased exposure to a particular olfactory stimulus (olfactory training) improves tolerance and pleasantness [34]. Although our study assessed only explicit sensory measures and did not evaluate the impact of implicit measures on the participant preferences, future studies assessing sense preferences should consider evaluating both implicit and explicit measures.

Our study is limited since it was performed in a single center and in a specific convenience sample of patients (children with sickle cell disease) and that may not be generalizable to all populations of patients. Moreover, our sample was dictated by time and budgetary constraints, rather than a formal sample size analysis, and, therefore, may lack appropriate power. Additionally, we did not attempt to compare acceptability of flaxseed to fish oil in this study, though one of our participants reported preference to the flaxseed-baked products to his daily fish oil capsule supplements.

## 5. Conclusions

Flaxseed is both acceptable and palatable to a cohort of children with sickle cell disease. Further trials comparing the efficacy and adherence of flaxseed-added products to fish oil supplements would be beneficial, as would longer-term research to test the potential anti-inflammatory effects of flaxseed supplementation on sickle-cell-disease-associated symptoms and pain.

## Data Availability

The data presented in this study are available on request from the corresponding author. The data are not publicly available due to ethical reasons.

## Supplementary Materials

The following supporting information can be downloaded at: https://www.mdpi.com/article/10.3390/nu15051245/s1, Document S1: Questionnaire used in the study, Document S2: Recipes for flaxseed baked products.
