# Supplementary material for: Palatability and Acceptability of Flaxseed-Supplemented Foods in Children with Sickle Cell Disease
Source: Nutrients. 2023 Mar 1;15(5):1245. doi: 10.3390/nu15051245 (PMC10004835; doi:10.3390/nu15051245)
Supplement: Supplementary file 1 [file nutrients-15-01245-s001.zip › nutrients-2220924-supplementary.pdf]

Supplemental content 1 (S1: Questionnaire)

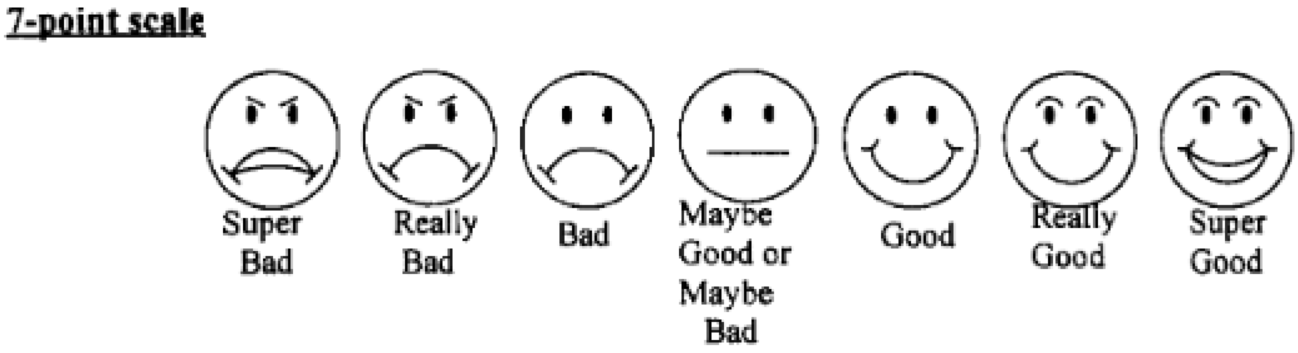

Please rate these products using the faces above based on the following

	Product 1 (Brownies)	Product 2 (Cookies)	Product 3 (Pancakes)	Product 4 (Applesauce)	Product 5 (Pudding)	Product 6 (Yogurt)
How does it look (1-7)<br>1 –Super bad (looks horrible/unpleasant)<br>7- Super good taste (looks appetizing)						
How does it smell (1-7)<br>1 –Super bad (horrible)<br>7- Super good						
How does it taste (1-7)<br>1 – Horrible/ super bad taste<br>7- Super good taste (I love it)						
How does it feel when I touch it (1-7)<br>1 – Super bad (I hate touching it)<br>7- Super good (I love touching it)						
How does it feel when I eat it (texture)<br>1--- Super bad (It is difficult to eat)<br>7— Super good (I love eating it)						
Favorite products (please pick 3) … I love it! (assign						

numbers to all products from 1-6<br>How often can you eat it? / For how long? (1 month, 2 months, 3 months…., 6 months, 1 year, many years)<br>Every day<br>Every other day<br>Twice / 3x a week<br>Once a week<br>Once a month<br>Other …………….						
Least favorite/ least liked products (please pick 3) --- I hate it!<br>Can you eat this product? / For how long? (1 month, 2 months, 3 months…., 6 months, 1 year, many years)<br>Every day<br>Every other day<br>Twice / 3x a week<br>Once a week<br>Once a month<br>Other…………………						
For older children 14 years and above						
How easy is it to follow the recipe and make it again (1-10)<br>1--- very difficult (I won't even try!)<br>10--- very easy (I can make it in a breeze)						
**PARENTS**						
How easy is it to follow the recipe and make it again (1-10)<br>1--- very difficult (I won't even try!)<br>10--- very easy (I can make it in a breeze)						

Is there anything we can do/add to make your least favorite product better? -------------------

Is there anything we can do/ add to make your favorite product even better? -------------------

Additional comments ---

May we contact you for the follow up study which would involve eating some of these products everyday as a snack for 4 weeks?

(i) Yes, I would like to participate
(ii) Maybe, I would like to be contacted but would decide at that time
(iii) No, I am not interested

If you are interested in the study, please pick one of the following:

(i) I would like the products premade and delivered to my house
(ii) I would like the products premade, and I will pick it up from the hospital
(iii) I would prefer to make it at home with the recipes

Age/ How old are you? …………………………………………….

Sex: Male/ Female? ……………………………………………

Do you live in a rural or urban setting?.......................................

THANK YOU!!!!

Supplemental content 2 (S2: Flaxseed)

FLAXSEED ADDED PRODUCTS RECIPES

BROWNIES

Flax Brownies
Ingredients
1 1/2 cups granulated sugar
3/4 cup all-purpose flour

2/3 cup cocoa powder, sifted if lumpy
1/2 cup powdered sugar, sifted if lumpy
1/2 cup dark chocolate chips
3/4 teaspoons sea salt
2 large eggs
1/2 cup canola oil
4 tablespoons water
1/2 teaspoon vanilla
180g Flax Meal

Instructions
Preheat the oven to 325°F. Lightly spray an 8x8 baking dish with cooking spray and line it with parchment paper. Spray the parchment paper.
In a medium bowl, combine the sugar, flour, cocoa powder, powdered sugar, chocolate chips, and salt.
In a large bowl, whisk together the eggs, oil, water, and vanilla.
Sprinkle the dry mix over the wet mix and stir until just combined. Add more water one tablespoon at a time if needed.
Pour the batter into the prepared pan (it'll be thick - that's ok) and use a spatula to smooth the top. Bake for 20 minutes, or until a toothpick comes out with only a few crumbs attached (note: it's better to pull the brownies out early than to leave them in too long). Cool completely before slicing. Store in an airtight container at room temperature for up to 3 days. These also freeze well!

COOKIES

Maple Cinnamon Cookies

COOKIE

1C unsalted butter
3/4C granulated sugar
3/4C brown sugar
1 egg
1tsp vanilla
1 2/3C AP flour
3/4tsp baking soda
1/4tsp baking powder
1/8tsp salt
270g golden flax seed meal

1.Cream butter and sugars together until well combined/almost fluffy
2.Add egg and vanilla- mix well

3.In a separate bowl whisk together all dry ingredients.
4.Add dry ingredients to butter mixture. Mix until everything is incorporated.
5. Portion dough by 1.5TBS (14g)
6. Chill 15-20 mins. Bake @350 for about 14 mins. rotating halfway.

GLAZE

1C powdered sugar
2tsp maple extract
About 2TBS hot water

Mix sugar, extract and 1TBS water together until smooth.
Add water as needed to reach desired consistency.

Makes 58 cookies
4.66g flax per cookie.

PANCAKES

1.Use box instructions for measuring pancake mix (any brand) for desired number of pancakes.
2. Multiply number of desired servings by 15 to determine quantity of flax. Use golden flax seed meal for best results.
3. Whisk together dry ingredients.
4.Use box instructions to measure water. Start with the amount noted for desired amount of servings.
5. For every cup of pancake mix, add 1 egg. Whisk is well into the water.
6. Combine wet and dry mixtures, add water as needed to achieve a smooth but not runny consistency.
7.Heat a large skillet or griddle over medium and rub with oil or cooking spray.
8.Scoop batter onto pan and top with sprinkles. Cook 2-3 minutes until golden, flip and cook another 2mins on the other side.
EXAMPLE:
1C Pearl Milling Brand pancake mix
60g golden flax
3/4water (to start)
1 egg
*Makes 8 pancakes. (4 servings) 4x15=60- The amount of golden flax seed meal needed.
*Can be made ahead, cooled and frozen for up to 2 mos.
